# First-Line Systemic Treatments of Metastatic Hormone-Sensitive Prostate Cancer: Updated Systematic Review and Network Meta-Analysis

**DOI:** 10.1097/JU.0000000000005131

**Published:** 2026-05-22

**Authors:** Keiichiro Miyajima, Marcin Miszczyk, Akihiro Matsukawa, Navid Roessler, Shota Inoue, Fumihiko Urabe, Keiichiro Mori, Takafumi Yanagisawa, Pierre I. Karakiewicz, Neeraj Agarwal, Alberto Briganti, Takahiro Kimura, Shahrokh F. Shariat

**Affiliations:** 1 Department of Urology, Comprehensive Cancer Center, Medical University of Vienna, Vienna, Austria; 2 Department of Urology, The Jikei University School of Medicine, Tokyo, Japan; 3 Collegium Medicum - Faculty of Medicine, WSB University, Dąbrowa Górnicza, Poland; 4 Department of Urology, University Medical Center Hamburg-Eppendorf, Hamburg, Germany; 5 Department of Urology, Okayama University Graduate School of Medicine, Dentistry and Pharmaceutical Sciences, Okayama, Japan; 6 Cancer Prognostics and Health Outcomes Unit, Division of Urology, University of Montréal Health Center, Montréal, Québec, Canada; 7 Huntsman Cancer Institute (NCI-CCC), University of Utah, Salt Lake City, Utah; 8 Unit of Urology/Division of Oncology, URI, IRCCS Ospedale San Raffaele, Milan, Italy; 9 Vita-Salute San Raffaele University, Milan, Italy; 10 Department of Urology, University of Texas Southwestern Medical Center, Dallas, Texas; 11 Hourani Center for Applied Scientific Research, Al-Ahliyya Amman University, Amman, Jordan; 12 Department of Urology, Weill Cornell Medical College, New York, New York; 13 Department of Urology, Semmelweis University, Budapest, Hungary; 14 Karl Landsteiner Institute of Urology and Andrology, Vienna, Austria; 15 College of Medical Health Technologies, Al-Turath University, Syndicates (Al-Niqabat Street), Mansour, Baghdad, Iraq

**Keywords:** prostatic neoplasms, neoplasm metastasis, drug therapy, survival rate, systematic review

## Abstract

**Purpose:**

The therapeutic landscape of metastatic hormone-sensitive prostate cancer (mHSPC) has expanded rapidly with the introduction of intensified combination regimens. The aim of this updated systematic review and network meta-analysis was to compare the efficacy and safety of first-line systemic combination therapies for mHSPC.

**Materials and Methods:**

We systematically searched MEDLINE, Embase, and Web of Science in March 2026 to identify randomized controlled trials evaluating first-line combination therapies for mHSPC. The outcomes of interest were progression-free survival and/or overall survival (OS) and severe adverse events. We conducted frequentist random-effects network meta-analyses, specifically limited to randomized controlled trials involving the all-comer population, and a systematic review for targeted and biomarker-driven strategies. The certainty of evidence (CoE) was assessed using the CINeMA (Confidence in Network Meta-Analysis) framework (PROSPERO: CRD420251161933).

**Results:**

Twenty-three trials (n = 18,689) were included. In the all-comer network meta-analysis, docetaxel + androgen receptor pathway inhibitor (ARPI) + androgen deprivation therapy (ADT; HR 0.66, 95% CI 0.43-1.01, CoE: moderate) and polymerase inhibitor (PARPi) + ARPI + ADT (HR 0.55, 95% CI 0.23-1.34, CoE: low) showed clinically relevant but not statistically significant progression-free survival improvements compared with ARPI + ADT. No triplet regimen demonstrated a statistically significant OS benefit over ARPI + ADT, although docetaxel + ARPI + ADT significantly improved OS in the high-volume subgroup (HR 0.76, 95% CI 0.61-0.95; CoE: high). While adding docetaxel or PARPi did not result in statistical significance for severe adverse event risk, the safety estimates were characterized by substantial imprecision, and a clinically meaningful increase in toxicity cannot be excluded. Overall, evidence certainty was downgraded because of risk of bias from open-label designs, clinical inconsistency in prior docetaxel exposure across comparator arms, and imprecision, particularly for PARPi-based regimens because of small sample sizes in phase II data. Targeted and biomarker-driven strategies (AMPLITUDE, CAPItello-281, and PSMAddition) further supported the benefit of intensification in selected or target-positive populations.

**Conclusions:**

Triplet intensification suggests a potential improvement in mHSPC, but OS benefits seem limited to specific subgroups, such as high-volume disease. The lack of a clear survival advantage in all-comers and the varying CoE support a selective, biomarker-driven or targeted approach. Treatment decisions should balance potential oncological gains against distinct toxicity profiles.

The therapeutic landscape for metastatic hormone-sensitive prostate cancer (mHSPC) has expanded substantially over the past decade. Current international guidelines recommend treatment intensification with androgen deprivation therapy (ADT) combined with an androgen receptor pathway inhibitor (ARPI), with or without docetaxel, as the standard of care for most patients.^1-3^ More recently, the AMPLITUDE, PSMAddition, and CAPItello-281 trials have introduced additional layers of complexity by demonstrating that incorporation of emerging agents—such as poly(ADP-ribose) polymerase inhibitors (PARPis), prostate-specific membrane antigen (PSMA)–directed theranostic therapies, and AKT inhibitors—may further improve clinically relevant outcomes.^4-6^ However, the relative magnitude of benefit among these increasingly diverse treatment strategies remains uncertain.

Our previous comparative analyses have helped clarify the benefit of established triplet therapy (docetaxel + ARPI + ADT) over conventional doublets, demonstrating improved survival outcomes and suggesting a potential advantage, particularly in patients with high-volume disease, although these findings were largely based on indirect comparisons and limited to established agents.^7-9^ Given the rapidly evolving evidence base, including the emergence of novel therapeutic strategies such as PARPi, PSMA-targeted radioligand therapy, and AKT inhibitors, a comprehensive synthesis is currently required to understand whether newer therapeutic combinations outperform existing standards. However, direct head-to-head comparisons among these increasingly diverse treatment strategies are lacking, precluding comprehensive comparisons using conventional pairwise meta-analysis. To address this gap, we performed an updated network meta-analysis (NMA) to synthesize survival outcomes from established therapies in the all-comer population, complemented by a systematic review (SR) of emerging targeted or biomarker-driven strategies, broadening our scope to include both phase II and phase III randomized evidence to capture the most recent therapeutic developments.

## METHODS

This SR and meta-analysis was prospectively registered in PROSPERO (CRD420251161933).

### Search Strategy

The review was conducted in accordance with PRISMA (Preferred Reporting Items for Systematic Reviews and Meta-Analyses) 2020 reporting standards and the PRISMA extension for reporting of SRs incorporating NMA^10,11^ and AMSTAR-2 guidelines^12^ (Supplementary Table 1–2; Appendix 1, https://www.jurology.com). We searched MEDLINE (through PubMed), Embase, and Web of Science Core Collection on March 20, 2026, for randomized trials evaluating systemic therapies for mHSPC. Full database-specific search strategies are available in Supplementary Appendix 2 (https://www.jurology.com). To ensure the comprehensiveness of our search, we manually screened the reference lists of all included studies and previously published SRs. In addition, we cross-referenced our findings with clinical trial registries (eg, ClinicalTrials.gov).

The primary outcome was progression-free survival (PFS). Secondary outcomes included overall survival (OS) and severe adverse events (AEs). Two reviewers independently screened titles/abstracts and full texts; discrepancies were resolved through consensus with additional coauthors.

### Inclusion and Exclusion Criteria

Eligible studies enrolled patients with mHSPC (P) treated with systemic combination therapies—including ADT, ARPIs, chemotherapy, or investigational agents (I)—compared with standard-of-care systemic regimens (C). Outcomes of interest were PFS, OS, and severe AEs (O). Only phase II and phase III randomized controlled trials (RCTs) were included to ensure the highest level of evidence and to minimize selection bias and confounding (S; PICOS framework).

We excluded studies that (1) exclusively enrolled patients with oligometastatic or bone-only disease treated primarily with local or metastasis-directed interventions, (2) evaluated bone-targeted or palliative treatments without systemic disease-modifying intent, (3) did not present original patient data (eg, reviews or commentaries), or (4) were not published in English. Conference abstracts were considered eligible if sufficient data were available for extraction.

### Data Extraction

Two authors independently extracted data using a standardized template, including first author, publication year, trial name and dates, sample size, patient age, proportion with de novo or high-volume disease, median follow-up, severe AE rates, and PFS/OS outcomes. When multiple PFS definitions were available, radiographic PFS (rPFS) was preferentially extracted; clinical PFS (cPFS) was accepted when rPFS was not reported. End points based solely on biochemical progression were excluded.

High-volume disease followed CHAARTED criteria—visceral metastases or ≥ 4 bone lesions with ≥ 1 beyond the vertebrae or pelvis.^13^ If alternative definitions were used, data were extracted as reported. Severe AEs were defined as grade ≥ 3 per National Cancer Institute CTCAE (Common Terminology Criteria for Adverse Events). For overlapping publications, the most complete dataset was selected. Any inconsistencies were resolved by consensus.

### Risk of Bias Assessment

Risk of bias in individual RCTs was evaluated independently by 2 reviewers for each specific outcome (PFS, OS, and severe AEs) using the Cochrane RoB 2 tool.^14^ Assessments were conducted only for those outcomes for which data were successfully extracted and included in the NMA or SR; outcomes for which data were not extracted from a given trial were not evaluated. Disagreements were resolved through discussion, with a third reviewer consulted when required. For bias due to missing outcome data, studies with less than 10% missing data and no imbalance between groups were considered low risk, whereas those with higher levels of missing data were judged as having some concerns or high risk, depending on the context.

### Network Construction

To preserve network connectivity, all ARPIs (abiraterone, apalutamide, darolutamide, enzalutamide, orteronel, and rezvilutamide) were grouped as a single class. ADT included medical or surgical castration, with or without first-generation antiandrogens. Regimens were categorized into 8 therapeutic classes:

(i) PARPi + ARPI + ADT
(ii) Docetaxel + ARPI + ADT
(iii) Immune checkpoint inhibitor + ARPI + ADT
(iv) Combination of 2 ARPI + ADT
(v) ARPI + ADT
(vi) Docetaxel + ADT
(vii) Cixutumumab + ADT
(viii) ADT

For the sensitivity analysis, we subdivided the ARPI class into 2 groups: androgen receptor antagonists (apalutamide, darolutamide, enzalutamide, and rezvilutamide) and CYP17 inhibitors (abiraterone and orteronel).

### Statistical Analysis

A frequentist random-effects NMA was performed using contrast-based inverse variance weighting to integrate direct and indirect evidence.^15,16^ Between-study variance (τ^2^) was estimated with the DerSimonian-Laird method. Transitivity assumptions were evaluated qualitatively by comparing eligibility criteria and key effect modifiers (age, de novo presentation, and disease volume) across trials. Subgroup NMAs were conducted for PFS and OS based on disease volume (high volume vs low volume). The credibility of subgroup effects was further evaluated using the ICEMAN (Instrument to Assess the Credibility of Effect Modification Analyses) tool for comparisons showing potential statistical interaction (*P* < .1).^17^ Sensitivity analyses were also performed by subdividing the ARPI group into AR antagonists and CYP17 inhibitors to assess the consistency of class-level effects. Log HRs and standard errors were derived from published HRs and 95% CIs.^18^ PFS analyses incorporated rPFS or, when unavailable, cPFS. Severe AEs were analyzed using arm-based risk ratios (RRs) with 95% CIs. Statistical significance was defined as a 2-sided *P* value of < .05. Clinically meaningful improvement was predefined as HR/RR of < 0.80 (representing a 20% relative reduction in risk), serving as an efficacy benchmark based on the American Society of Clinical Oncology perspective.^19^ To facilitate clinical interpretation, absolute differences in 3-year PFS and OS rates were estimated by applying the NMA-derived HRs to assumed baseline survival rates for the reference treatment (ARPI + ADT). The baseline 3-year rates for ARPI + ADT were established at 0.63 for PFS and 0.78 for OS, based on ARCHES trials.^20^ By contrast, absolute risks for AEs were not estimated because applying the RRs to baseline risks resulted in values exceeding 100% in certain arms. Thus, safety outcomes were reported exclusively as RRs.

Statistical heterogeneity was assessed with Cochran Q test (*P* < .05 considered significant). When heterogeneity was detected, leave-one-out sensitivity analyses were conducted. Treatment rankings were estimated using P-scores.^21^ All analyses were performed in R (version 4.5.0) using the “meta” and “netmeta” packages. Ethical approval was not required because only published aggregate data were used. This study was conducted as an independent academic project without any external funding or involvement from commercial entities. The research process, including study selection, data extraction, and interpretation of the findings, was managed strictly by the authors to prevent any potential conflicts of interest from influencing the results.

### Certainty of Evidence Assessment

The certainty of evidence (CoE) was evaluated using the CINeMA (Confidence in Network Meta-Analysis) framework and completed for the direct comparisons, indirect comparisons, and overall network comparisons.^22,23^ If meaningful incoherence between direct and indirect evidence was identified, the estimate with the higher certainty and statistical precision would be preferred.

Confidence was assessed across 6 domains: within-study bias, reporting bias, indirectness, imprecision, heterogeneity, and incoherence. Reporting bias was assessed by comparing the outcomes predefined in trial protocols or registries (eg, ClinicalTrials.gov) with those reported in the published articles. For comparisons involving 10 or more studies, publication bias was further evaluated using funnel plots and Egger test. For imprecision assessment, a threshold of 1.2 (HR or RR) was predefined, aligning with the GRADE minimally contextualized approach.^24^ CoE ratings were downgraded by 1 level for “some concerns” and 2 levels for “major concerns” based on an integrated assessment of these interconnected domains, rather than simple arithmetic summation.

## RESULTS

### Study Selection and Characteristics

**Figure 1.**
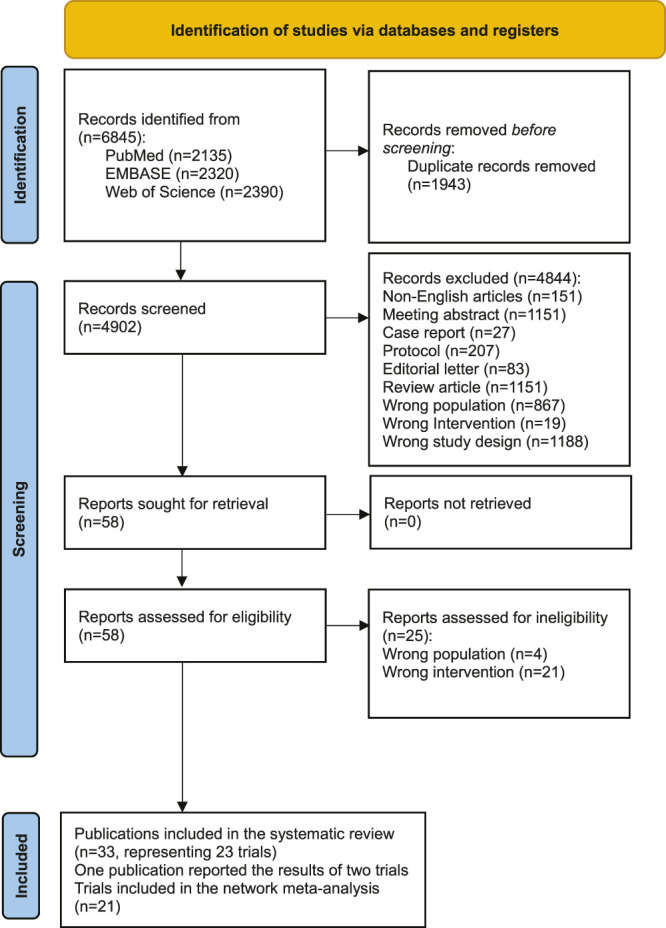
PRISMA (Preferred Reporting Items for Systematic Reviews and Meta-Analyses) 2020 flow diagram. Twenty-three trials (n = 18,89) met inclusion criteria after screening and eligibility assessment.

Twenty-three RCTs met the inclusion criteria: 18 phase III RCTs (n = 18,058)^4-6,13,20,25-46^ and 5 phase II RCTs (n = 631; Table 1),^47-52^ corresponding to 33 unique studies. One publication included 2 separate RCTs.^35^ The PRISMA flow diagram is shown in Figure 1. The studies excluded at the full-text screening were summarized in the Supplementary Table 3 (https://www.jurology.com).

**Table 1.** Basic Characteristics of the Included Randomized Controlled Trials

Trial, author, year	Period	Tx arm	Ctrl arm	Inclusion criteria	Imaging for diagnosis	Funding
Phase III						
AMPLITUDE Attard et al,^4^ 2025	Dec 2020-Jul 2023	Nira + Abi + ADT	Pbo + Abi + ADT	mHSPC with HRR gene alterations	Bone scan, CT, MRI	Johnson & Johnson
ARANOTE Saad et al,^43^ 2024	Mar 2021- Aug 2022	Daro + ADT	Pbo + ADT	mHSPC	Bone scan, CT, MRI	Bayer and Orion Pharma
ARASENS Smith et al,^44^ 2022	Nov 2016-Jun 2018	Daro + Doc + ADT	Pbo + Doc + ADT	mHSPC	Bone scan, CECT, MRI	Bayer and Orion Pharma
ARCHES Armstrong et al,^25^ 2019 Armstrong et al,^20^ 2022 Armstrong et al,^34^ 2026	Mar 2016-Jan 2018	Enz + ADT	Pbo + ADT	mHSPC	Bone scan, CT, MRI	Astellas Pharma Inc and Pfizer Inc
CAPItello-281 Fizazi et al,^6^ 2025	Jul 2022-Feb 2024	Capi + Abi + ADT	Pbo + Abi + ADT	De novo mHSPC with PTEN deficiency	CT, MRI	AstraZeneca
CHAARTED Sweeney et al,^32^ 2015 Kyriakopoulos et al,^13^ 2018 Tripathi et al,^46^ 2025	Jul 2006-Dec 2012	Doc + ADT	ADT	mHSPC	Bone scan, CT	NCI/NIH; Sanofi
CHART Gu et al,^42^ 2022	Jun 2018-Aug 2020	Rez + ADT	NSAA + ADT	High-volume mHSPC	Bone scan, CT, MRI	Jiangsu Hengrui Pharmaceuticals
ENZAMET Davis et al,^27^ 2019 Sweeney et al,^45^ 2023	Mar 2014-Mar 2017	Enz + ADT	NSAA + ADT	mHSPC	Bone scan, CT	Astellas Pharma
GETUG-AFU15 Gravis et al,^29^ 2013 Gravis et al,^41^ 2016	Oct 2004-Dec 2008	Doc + ADT	ADT	mHSPC	Bone scan, CT	UNICANCER, PHRC, INCa, Sanofi-Aventis, AstraZeneca, Amgen
KEYNOTE-991 Gratzke et al,^40^ 2025	Mar 2020-Aug 2021	Pem + Enz + ADT	Enz + ADT	mHSPC	CT, MRI	Merck Sharp & Dohme LLC
LATITUDE Fizazi et al,^28^ 2017 Fizazi et al,^39^ 2019	Feb 2013-Dec 2014	Abi + ADT	Pbo + ADT	High-risk mHSPC	Bone scan, CT, MRI	Janssen Research & Development
PEACE-1 Fizazi et al,^38^ 2022	Nov 2013-Dec 2018	Abi + Doc + ADT	Pbo + Doc + ADT	De novo mHSPC	Bone scan, CT, MRI	Janssen-Cilag, Ipsen, Sanofi, and the French government
PSMAddition Tagawa et al,^5^ 2025	NR	Lu-PSMA+ARPI+ADT	ARPI + ADT	PSMA PET–positive mHSPC	PSMA PET/CT	Novartis Pharmaceuticals
STAMPEDE (arm B, C, E) James et al,^31^ 2016 Clarke et al,^37^ 2019	Oct 2005-Mar 2013	Doc + ADT	ADT	mHSPC	Bone scan, CT, MRI	Cancer Research UK, Medical Research Council, Astellas, Clovis Oncology, Janssen, Novartis Pfizer and Sanofi-Aventis
STAMPEDE (arm G) James et al,^30^ 2017 Attard et al,^35^ 2023	Oct 2011-Jan 2014	Abi + ADT	ADT	mHSPC	Bone scan, CT, MRI	Cancer Research UK, UK Medical Research Council, Janssen, Astellas, and Swiss Group for Clinical Cancer Research
STAMPEDE (arm J) Attard et al,^35^ 2023	Jul 2014-Mar 2016	Abi + Enz + ADT	ADT	mHSPC	Bone scan, CT, MRI	Cancer Research UK, UK Medical Research Council, Janssen, Astellas, and Swiss Group for Clinical Cancer Research
SWOG-1216 Agarwal et al,^33^ 2022	Mar 2013-Jul 2017	Ort + ADT	NSAA + ADT	mHSPC	Bone scan, CT, MRI	NIH/NCI; Millennium Pharmaceuticals (Takeda)
TITAN Chi et al,^26^ 2019 Chi et al,^36^ 2021	Dec 2015- Jul 2017	Apa + ADT	Pbo + ADT	mHSPC	Bone scan, CT, MRI	Janssen Research & Development
Phase II						
Lou et al,^49^ 2024	Jun 2018-Jun 2021	Abi + ADT	ADT	mHSPC	NR	Academic (institutional funding only)
NCT02058706 Vaishampayan et al,^51^ 2021	NR	Enz + ADT	NSAA + ADT	mHSPC	Bone scan, CT	NIH; Astellas Pharma Global Development, Inc
NCT02059213 Palmbos et al,^48^ 2021	Jul 2014-Feb 2017	Palbo + ADT	ADT	mHSPC with RB positive	Bone scan, CT, MRI	Pfizer with additional academic/public grants
SWOG S0925 Yu et al,^47^ 2015 Wong et al,^52^ 2020	NR	Cix + NSAA + ADT	NSAA + ADT	mHSPC	Bone scan, CT, MRI	NCI/NIH; Fred Hutchinson Cancer Research Center, ImClone Systems
ZZFIRST Mateo et al,^50^ 2024	NR	Tara + Enz + ADT	Enz + ADT	High-volume mHSPC	Bone scan, CT, MRI	MedSIR

Abbreviations: Abi, abiraterone acetate; ADT, androgen deprivation therapy; Apa, apalutamide; ARPI, androgen receptor pathway inhibitor; Capi, capivasertib; CECT, contrast-enhanced CT; Cix, cixutumumab; Ctrl, control; Daro, darolutamide; Doc, docetaxel; Enz, enzalutamide; Lu-PSMA, 177Lu-PSMA-617; mHSPC, metastatic hormone-sensitive prostate cancer; NCI, National Cancer Institute; Nira, niraparib; NR, not reported; NSAA, nonsteroidal antiandrogen; Ort, orteronel; Palbo, palbociclib; Pbo, placebo; Pem, pembrolizumab; PET, positron emission tomography; PSMA, prostate-specific membrane antigen; Rez, rezvilutamide; Tara, tarazoparib; Tx, treatment.

Baseline characteristics are summarized in Table 2. Median age ranged from 63 to 70 years, and median follow-up duration from 21.1 to 120 months. Across studies, the proportion of de novo metastatic disease ranged from 48% to 100% and high-volume disease from 41% to 100%. Two trials categorized metastatic burden into minimal (vertebral/pelvic bone and/or lymph node involvement) and extensive disease (any spread beyond minimal criteria).^33,48^

**Table 2.** Patient Characteristics and Outcomes of the Included Randomized Controlled Trials

Trial	N		Age, median (IQR), y; or mean ± SD		De novo (%)		High-volume (%)			HR (95% CI) for PFS	HR (95% CI) for OS	Median follow-up (months)
	Tx arm	Ctrl arm	Tx arm	Ctrl arm	Tx arm	Ctrl arm	Tx arm	Ctrl arm				
Phase III												
AMPLITUDE	348	348	68 (40-88)	67 (40-92)	87	87	77	78	rPFS	0.63 (0.49-0.80)	0.79 (0.55-1.04)	30.8
ARANOTE	446	223	70 (43-93)	70 (45-91)	71	75	71	70	rPFS	0.54 (0.41-0.71)	0.81 (0.59-1.12)	25.1
ARASENS	651	655	67 (41-89)	67 (42-86)	86	87	76	78	NR	NR	0.68 (0.57-0.80)	43
ARCHES	574	576	70 (46-92)	70 (42-92)	70	63	62	65	rPFS	0.63 (0.52-0.76)	0.7 (0.58-0.85)	61.4
CAPItello-281	507	505	67 (2-87)	68 (43-88)	100	100	74	75	rPFS	0.81 (0.66-0.98)	0.9 (0.71-1.15)	33.2/25.7
CHAARTED	397	393	64 (36-88)	63 (39-91)	73	73	66	64	cPFS	0.62 (0.51-0.75)	0.78 (0.66-0.93)	120
CHART	326	328	69 (64-74)	69 (64-75)	NR	NR	100	100	rPFS	0.44 (0.33-0.58)	0.58 (0.44-0.77)	29.3
ENZAMET	563	562	69 (63-74)	69 (64-75)	62	60	53	54	cPFS	0.45 (0.39-0.53)	0.7 (0.58-0.84)	68
GETUG-AFU15	192	193	63 (57-68.2)	64 (58-70)	67	76	48	47	rPFS	0.69 (0.55-0.87)	0.88 (0.68-1.14)	83.9
KEYNOTE-991	626	625	68 (43-91)	68 (37-90)	NR	NR	63	64	rPFS	1.20 (0.96-1.49)	1.16 (0.88-1.53)	21.1
LATITUDE	597	602	67.3 ± 8.5	66.8 ± 8.7	100	100	82	78	rPFS	0.47 (0.39-0.55)	0.66 (0.56-0.78)	51.8
PEACE-1	355	355	66 (37-85)	66 (44-84)	100	100	63	65	rPFS	0.50 (0.34-0.71)	0.75 (0.59-0.95)	45.7
PSMAddition	572	572	68 (38-91)	68 (36-90)	52	48	68	68	rPFS	0.72 (0.58-0.9)	0.84 (0.63-1.13)	23.6
STAMPEDE (arm B, C, E)	362	724	65 (60-70)	65 (60-71)	96	95	44	41	PFS	0.69 (0.59-0.81)	0.81 (0.69-0.95)	78.2
STAMPEDE (arm G)	501	502	67 (42-85)	67 (39-84)	93	95	51	54	PFS	0.47 (0.4-0.55)	0.62 (0.53-0.73)	96
STAMPEDE (arm J)	462	454	69 (48-84)	68 (37-83)	93	94	46	42	PFS	0.5 (0.42-0.59)	0.65 (0.55-0.77)	72
SWOG-1216	638	641	68 (46-90)	68 (19-92)	NR	NR	49^a^	49^a^	PFS	0.58 (0.51-0.67)	0.86 (0.72-1.02)	70.8
TITAN	525	527	69 (45-94)	68 (43-90)	78	84	62	64	rPFS	0.48 (0.39-0.60)	0.65 (0.53-0.79)	44
Phase II												
Lou et al^49^	118	118	76 ± 8	76 ± 8	NR	NR	NR	NR	NR	NR	NR	24
NCT02058706	36	35	66 (54-86)	63 (51-84)	NR	NR	56	49	NR	NR	0.31 (0.13-0.74)	39
NCT02059213	40	20	68 (47-87)	66 (44-81)	NR	NR	60^a^	58^a^	NR	NR	NR	NR
SWOG S0925	105	105	65 (60-72)	66 (58-73)	NR	NR	NR	NR	rPFS	1.17 (0.85-1.6)	1.01 (0.7-1.45)	63.6
ZZFIRST	37	17	68 (47-86)	69 (55-82)	97	100	100	100	rPFS	0.55 (0.23-1.33)	NR	30.6

Abbreviations: cPFS, clinical progression-free survival; Ctrl, control; NR, not reported; OS, overall survival; PFS, progression-free survival; rPFS, radiographic progression-free survival; Tx, treatment.

a A disease volume was defined as follows: Minimal disease was limited to involvement of the vertebrae and/or pelvic bones and/or lymph nodes; extensive disease was defined as any involvement greater than minimal. We extracted data for extensive disease.

### Risk of Bias and Study Quality

Risk of bias was evaluated separately for each outcome (Supplementary Table 4, https://www.jurology.com). For PFS, 9 trials were rated as low risk and 10 as having some concerns. For OS, 19 trials rated low risk and one as having some concern. For severe AEs, 9 were rated as low risk and 6 as having some concerns. The primary driver for some concerns was the open-label design of several trials, which may have introduced bias in outcome assessment (eg, investigator-assessed progression) and patient-reported outcomes such as AEs. Minor concerns regarding missing outcome data were also noted in a small number of studies.

### NMA in the All-Comer Population

The all-comer network was well connected, centered on ADT and ARPI + ADT. No major transitivity violations were observed across key modifiers, including age, disease volume, and de novo status.

#### PFS

**Figure 2.**
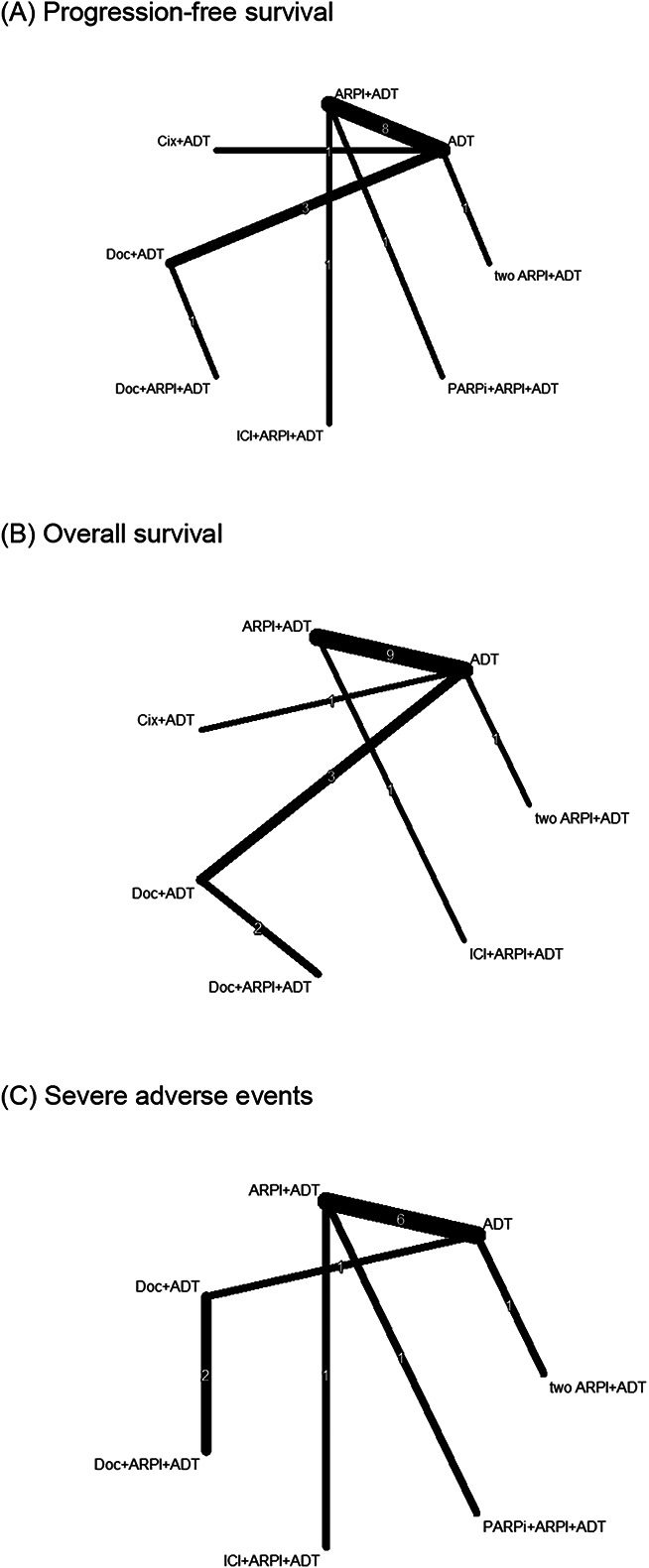
Network plots of the network meta-analyses of treatment regimens for metastatic hormone-sensitive prostate cancer. A, Progression-free survival. B, Overall survival. C, Severe adverse events. ADT indicates androgen deprivation therapy; ARPI, androgen receptor pathway inhibitor; Cix, cixutumumab; Doc, docetaxel; ICI, immune checkpoint inhibitor; PARPi, poly(ADP-ribose) polymerase inhibitor.

Sixteen trials (n = 13,533) reported PFS^33-43,45,46,50,51^ (network structure shown in Figure 2, A). Eight trials (n = 8131) compared ARPI + ADT vs ADT,^33-36,39,42,43,45^ and 3 trials (n = 2261) compared docetaxel + ADT with ADT.^37,41,46^ No significant heterogeneity was observed in either analysis (Supplementary Figure 1, https://www.jurology.com).

**Figure 3.**
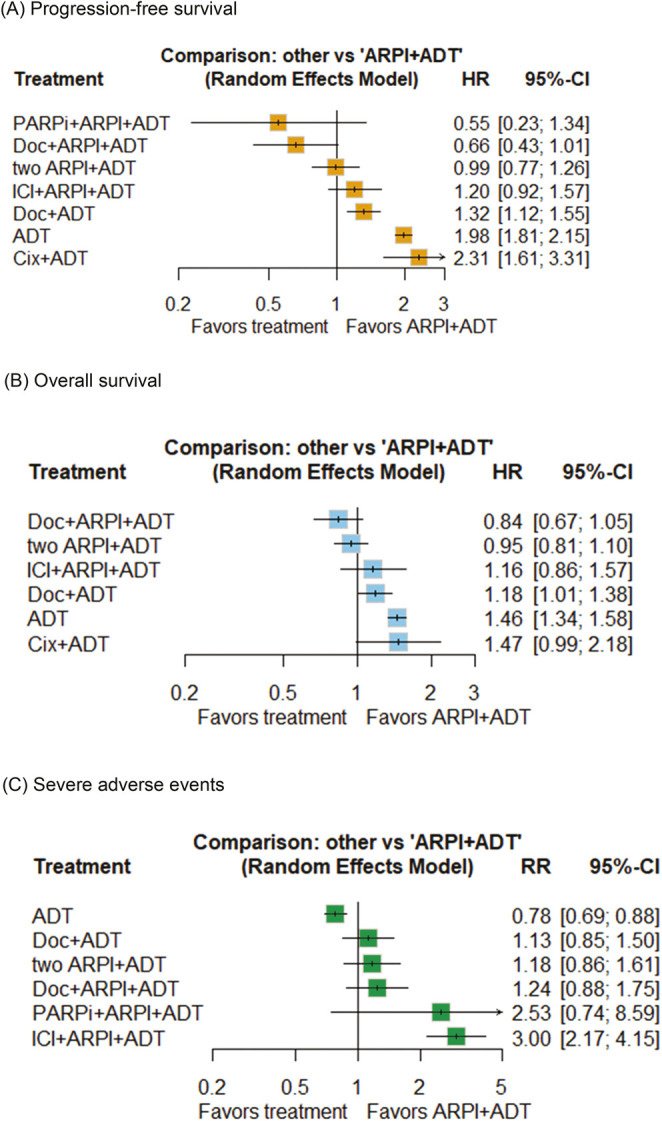
Forest plot of the primary network meta-analyses of treatment regimens for metastatic hormone-sensitive prostate cancer. A, Progression-free survival. B, Overall survival. C, Severe adverse events. ADT indicates androgen deprivation therapy; ARPI, androgen receptor pathway inhibitor; Cix, cixutumumab; Doc, docetaxel; ICI, immune checkpoint inhibitor; PARPi, poly (ADP-ribose) polymerase inhibitor; RR, risk ratio.

Classified treatment comparisons are shown in Figure 3, A. Compared with ARPI + ADT, PARPi + ARPI + ADT (HR 0.55, 95% CI 0.23-1.34, *P* = .2, CoE: low, P-score: 0.91) and docetaxel + ARPI + ADT (HR 0.66, 95% CI 0.43-1.01, *P* = .056, CoE: moderate, P-score: 0.90) showed a clinically relevant effect size, although they did not reach statistical significance. In absolute terms, these improvements translated into an estimated 15% increase in the 3-year PFS rate (95% CI −9.1% to 27%) for PARPi + ARPI + ADT and an 11% increase (95% CI −0.3% to 19%) for docetaxel + ARPI + ADT. No other regimen showed a clinically meaningful benefit compared with ARPI + ADT. Furthermore, network comparisons among these triplet combinations and ARPI + ADT identified no statistically significant differences (Supplementary Table 5, https://www.jurology.com). P-score rankings were generally consistent with these findings (Table 3), and there was no evidence of significant heterogeneity in the NMA. Sensitivity analyses yielded results consistent with the primary analysis (Supplementary Figure 2, Supplementary Table 6, https://www.jurology.com).

**Table 3.** Ranking of Treatments Based on P-Scores

NMA	Summary of included studies	Treatment ranking
PFS		
Overall	16 trials	PARPi + ARPI + ADT: 91% > Doc + ARPI + ADT: 90% > 2 ARPI + ADT: 65% >ARPI + ADT: 64% > ICI + ARPI + ADT: 43% > Doc + ADT: 33% > ADT: 12%> Cix + ADT: 3%
	8 comparisons	
	13,553 patients	
High-volume	12 trials	Doc + ARPI + ADT: 90% > PARPi + ARPI + ADT: 84% > ARPI + ADT: 55% > Doc + ADT: 44% > ICI + ARPI + ADT: 24% > ADT: 3%
	6 comparisons	
	6348 patients	
Low-volume	10 trials	ICI + ARPI + ADT: 76% >Doc + ARPI + ADT: 70% > ARPI + ADT: 68% > Doc + ADT: 30% > ADT: 6%
	5 comparisons	
	3649 patients	
OS		
Overall	17 trials	Doc + ARPI + ADT: 95% > 2 ARPI + ADT: 79% >ARPI + ADT: 68% > ICI + ARPI + ADT: 44% > Doc + ADT: 39% > Cix + ADT: 14% > ADT: 10%
	7 comparisons	
	14,885 patients	
High-volume	16 trials	Doc + ARPI + ADT: 99% > ARPI + ADT: 70% > 2 ARPI + ADT: 66% >Doc + ADT: 51% > ICI + ARPI + ADT: 46% > ADT: 18% > Cix + ADT: 0%
	7 comparisons	
	9060 patients	
Low-volume	14 trials	ARPI: 84% > 2 ARPI + ADT: 67% > Doc + ARPI + ADT: 62% > ICI + ARPI + ADT: 58% > Doc + ADT: 22% > ADT: 7%
	6 comparisons	
	5155 patients	
Severe AEs		
Overall	12 trials	ADT: 99% > ARPI + ADT: 74% > Doc + ADT: 59% > 2 ARPI + ADT: 51% > Doc + ARPI + ADT: 42% > PARPi + ARPI + ADT: 18% > ICI + ARPI + ADT: 7%
	7 comparisons	
	12,161 patients	

Abbreviations: ADT, androgen deprivation therapy; AE, adverse event; ARPI, androgen receptor pathway inhibitor; Cix, cixutumumab; Doc, docetaxel; ICI, immune checkpoint inhibitor; NMA, network meta-analysis; OS, overall survival; PARPi, poly (ADP-ribose) polymerase inhibitor; PFS, progress-free survival.

#### OS

Seventeen trials (n = 14,885) reported OS^33-46,51,52^ (network structure shown in Figure 2, B). Nine trials (n = 8202) compared ARPI + ADT vs ADT,^33-36,39,42,43,45,51^ 3 trials (n = 2261) compared docetaxel + ADT vs ADT,^37,41,46^ and 2 trials (n = 2015) compared docetaxel + ARPI + ADT vs docetaxel + ADT.^38,44^ No significant heterogeneity was observed (Supplementary Figure 3, https://www.jurology.com).

Compared with ARPI + ADT, docetaxel + ARPI + ADT (HR 0.84, 95% CI, 0.67-1.05, *P* = .1, CoE: moderate, P-score: 0.95) did not achieve statistical significance or a clinically meaningful improvement in OS (Figure 3, B).

In absolute terms, this translated into an estimated 3.2% increase in the 3-year OS rate (95% CI −1.0% to 6.8%). Similarly, no other regimens showed a clinically meaningful benefit over ARPI + ADT (Supplementary Table 7, https://www.jurology.com). PARPi + ARPI + ADT could not be evaluated for OS because relevant data were not available from the included clinical trials.

P-score rankings were generally consistent with these findings (Table 3), and there was no evidence of significant heterogeneity in the NMA. Furthermore, sensitivity analyses yielded results consistent with the primary analysis (Supplementary Figure 4, Supplementary Table 8, https://www.jurology.com).

#### Severe Adverse Event

Twelve trials (n = 12,161)^34-40,43-45,50^ reported severe AEs (network structure shown in Figure 2, C). Six trials (n = 6183) compared ARPI + ADT vs ADT,^34-36,39,43,45^ and 2 trials (n = 1999) compared docetaxel + ARPI + ADT vs docetaxel + ADT.^38,44^ No significant heterogeneity was identified (Supplementary Figure 5, https://www.jurology.com). Although docetaxel + ARPI + ADT (RR 1.24, 95% CI, 0.88-1.75, *P* = .2, CoE: moderate, P-score: 0.42) and PARPi + ARPI + ADT (RR 2.53, 95% CI 0.74-8.59, *P* = .1, CoE: low, P-score: 0.18) did not achieve statistical significance compared with ARPI + ADT, both showed a numerical increase in the risk of severe AEs that may be clinically meaningful. By contrast, only ADT monotherapy (RR 0.78, 95% CI, 0.69-0.88, *P* < .001, CoE: moderate, P-score: 0.99) demonstrated a clinically and statistically significant reduction in risk relative to ARPI + ADT (Figure 3, C).

Although no statistically significant difference was identified between the 2 triplet regimens, the numerical disparity in point estimates suggests a higher risk of severe AEs for PARPi + ARPI + ADT compared with docetaxel + ARPI + ADT (RR 2.03, 95% CI 0.57-7.24, CoE: low; Supplementary Table 9, https://www.jurology.com). P-score rankings were generally consistent with these findings (Table 3), and there was no evidence of significant heterogeneity in the NMA. Furthermore, sensitivity analyses yielded results consistent with the primary analysis (Supplementary Figure 6, Supplementary Table 10, https://www.jurology.com).

#### Certainty of Evidence

Supplementary Table 11 (https://www.jurology.com) presents the CoE assessment. In our study, because the treatment network did not contain closed loops for all comparisons, statistical incoherence was generally nonapplicable; in such cases, incoherence was judged as “no concern” and the network estimate was used. Regarding reporting bias, no strong evidence of publication bias was identified through registry cross-referencing.

For PFS, most comparisons were rated as moderate certainty. In addition to within-study bias and incoherence, downgrading was also applied for indirectness in certain comparisons to account for the clinical heterogeneity of comparator arms (eg, varying exposure to docetaxel). Comparisons involving PARPi-based regimens were rated as low certainty, primarily because of imprecision. For OS, the CoE was generally high, although some comparisons involving ARPI + ADT vs docetaxel-based regimens were downgraded to moderate certainty because of heterogeneity and/or imprecision. For severe AEs, most comparisons were of moderate certainty, while PARPi-based comparisons were rated as low certainty, mainly because of imprecision.

#### Subgroup Analysis by Disease Volume

Subgroup networks are shown in Supplementary Figure 7 (https://www.jurology.com).

##### High-Volume Disease

For PFS, 12 trials (n = 6348)^33,36-43,45,46,50^ were included. Compared with ARPI, both docetaxel + ARPI + ADT (HR 0.51, 95% CI 0.26-1.02, *P* = .06, CoE: low, P-score: 0.9) and PARPi + ARPI + ADT (HR 0.55, 95% CI 0.21-1.45, *P* = .2, CoE: very low, P-score: 0.84) demonstrated a clinically relevant effect size, although neither reached statistical significance (Supplementary Figure 8A, https://www.jurology.com). Substantial heterogeneity in the NMA was observed (I^2^ = 80%, *P* < .001), which served as a primary factor for downgrading the CoE (Supplementary Table 12, https://www.jurology.com). P-score rankings followed a similar trend (Table 3).

For OS, 16 trials (n = 9060)^33-46,52^ were included. Compared with ARPI + ADT, docetaxel + ARPI + ADT (HR 0.76, 95% CI 0.61-0.95, *P* = .01, CoE: high, P-score: 0.99) demonstrated both clinical and statistical significance (Supplementary Figure 8B, https://www.jurology.com). No evidence of significant heterogeneity was observed within the NMA.

##### Low-Volume Disease

For PFS, 10 trials (n = 3649) were included.^33,36-41,43,45,46^ No treatment regimen demonstrated a clinically or statistically significant improvement over ARPI + ADT, although ARPI + ADT remained superior to ADT alone (Supplementary Figure 9A, https://www.jurology.com).

For OS, 14 trials (n = 5155) were included.^33-41,43-46^ No treatment regimen demonstrated a clinically or statistically significant improvement over ARPI + ADT, although ARPI + ADT demonstrated a clinically and statistically significant improvement over ADT alone and docetaxel + ADT (Supplementary Figure 9B, https://www.jurology.com).

##### Credibility of Subgroup Effects

Exploratory interaction analyses suggested a potential effect modification by disease volume for docetaxel-based regimens. Application of the ICEMAN tool indicated moderate credibility for these subgroup effects (Supplementary Table 13, https://www.jurology.com).

### SR of Emerging Targeted or Biomarker-Driven Strategies

Owing to the distinct study populations defined by specific precision medicine criteria, the following trials were evaluated qualitatively rather than being integrated into the all-comer NMA.

#### AMPLITUDE Trial (PARP Inhibition)

In the AMPLITUDE trial,^4^ conducted in patients with HRR-mutated mHSPC, the addition of niraparib to abiraterone achieved both statistical and clinical significance in improving rPFS (HR 0.63, 95% CI 0.49-0.80). Although the point estimate for OS was favorable (HR 0.79, 95% CI 0.59-1.04), the data remain immature. However, severe AEs were substantially increased (75% vs 59%), primarily driven by anemia (29%) and hypertension (26%), with a high rate of transfusion requirement.

#### CAPItello-281 Trial (AKT Inhibition for PTEN-Deficient Disease)

In the CAPItello-281 trial,^6^ conducted in patients with PTEN-deficient mHSPC, the addition of capivasertib to abiraterone resulted in a statistically significant improvement in rPFS (HR 0.81, 95% CI 0.66-0.98). By contrast, OS data remain immature (HR 0.9, 95% CI 0.71-1.15). Severe AEs were substantially increased (67% vs 40%), mainly driven by AKT inhibitor–related toxicities such as rash (12%) and hyperglycemia (10%).

#### PSMAddition Trial (Radioligand Therapy)

In the PSMAddition trial,^5^ the addition of 177 Lu-PSMA to ARPI + ADT achieved both statistical and clinical significance in improving rPFS (HR 0.72, 95% CI 0.58-0.90). OS data remain immature (HR 0.84, 95% CI 0.63-1.13). Severe AEs were modestly increased (51% vs 43%), with a safety profile consistent with radioligand therapy, including cytopenias and hypertension.

## DISCUSSION

This study provides a comprehensive evaluation of the rapidly evolving mHSPC treatment landscape by integrating an updated NMA of all-comer populations with an SR of the latest biomarker-driven and targeted strategies.

In the NMA for the all-comer population, docetaxel + ARPI + ADT demonstrated a clinically relevant effect size for rPFS compared with ARPI + ADT, although it did not reach statistical significance, with moderate CoE. PARPi + ARPI + ADT showed a similar trend, but with low certainty. For OS, no regimen significantly improved outcomes compared with ARPI + ADT. However, in the high-volume subgroup, docetaxel + ARPI + ADT demonstrated both clinically and statistically significant benefit with high certainty. Regarding safety, ADT monotherapy was the only regimen associated with a significantly lower risk of severe AEs, whereas other regimens tended to increase risk compared with ARPI + ADT.

Consistent with these findings, targeted and biomarker-driven trials showed that adding targeted agents to ARPI + ADT improves rPFS in selected populations, although OS data remain immature. These intensified strategies were also associated with increased toxicity.

A previous living NMA has evaluated first-line treatment options for mHSPC.^53^ However, our study extends this work by incorporating more recent phase trials and emerging treatment strategies, including targeted or biomarker-driven approaches, and by applying CINeMA, thereby providing a more comprehensive and contemporary assessment of available treatment options.

In our NMA, docetaxel + ARPI + ADT demonstrated a clinically relevant effect size for rPFS compared with ARPI + ADT. However, unlike our previous NMA^7^ based on subgroup data from ARCHES,^20^ ENZAMET,^45^ and TITAN,^36^ we did not observe a statistically significant improvement in PFS. The discrepancy likely reflects methodological differences: The previous analysis separated trials by prior docetaxel exposure, whereas the present NMA grouped regimens into broader categories, acknowledging the substantial heterogeneity in exposure timing and coadministration across trials. Interpretation of this result is supported by moderate CoE, indicating a reasonable level of confidence in the estimate. Although the observed risk reduction with docetaxel + ARPI + ADT remains substantial, the statistical precision was likely attenuated by the inherent heterogeneity of an all-comer population. This reflects a dilution of the effect size when transitioning from the highly selected subgroups of our previous NMA to the broader cohort analyzed in the present NMA**.**

Importantly, in the high-volume subgroup, docetaxel + ARPI + ADT achieved both clinically and statistically significant benefit in OS compared with ARPI + ADT with high CoE. This finding differs from our previous NMA and may be partly explained by updated OS data from key trials, including ARCHES^34^ and CHAARTED,^46^ which likely influenced the indirect estimates within the high-volume network. Given the high CoE, these findings support the use of docetaxel-based intensification in patients with high-volume disease.

Similarly, PARPi + ARPI + ADT showed a clinically relevant effect size but not statistical significance in rPFS with low CoE. This finding was primarily informed by early data from the phase II ZZFIRST trial,^50^ which suggested a potential benefit in high-volume mHSPC. By contrast, results from the AMPLITUDE trial demonstrated that adding niraparib to abiraterone + ADT significantly reduced PFS by 37% compared with ARPI + ADT in HRR-mutated mHSPC cohorts. HRR-mutated and all-comer mHSPC cohorts differ markedly in biology and prognosis, and evidence from metastatic castration-resistant prostate cancer demonstrates that PARPi + ARPI improves outcomes only in HRR-mutated disease.^54^ Approximately 20% of men with mHSPC harbor HRR alterations,^55^ and early identification is critical, yet germline testing uptake remains low even in metastatic castration-resistant prostate cancer.^56^ Ongoing studies—including TALAPRO-3^57^ and EvoPAR-Prostate01^58^—will clarify the role of PARPi combinations in both HRR-mutated and nonmutated mHSPC.

Careful biomarker-driven patient selection is also essential when considering AKT inhibition in mHSPC. PTEN loss is observed in approximately 25 to 40% of patients and is associated with poor prognosis.^6,59^ In this context, the CAPItello-281 trial demonstrated a 19% reduction in the risk of progression, suggesting potential benefit in this biomarker-defined population. Similar to HRR alterations, early identification of PTEN loss may be important for optimal treatment selection. However, this approach is associated with increased toxicity, highlighting the need for careful AE management in clinical practice.

The PSMAddition trial showed that Lu-177–PSMA + ARPI + ADT reduced PFS by 28% compared with ARPI + ADT. Given that approximately 90% of patients with mHSPC have PSMA-positive disease,^60^ this strategy could be applied to a large proportion of the population. However, evidence is currently limited to a single interim analysis, and the final trial results are eagerly awaited. The safety profile was manageable, with hypertension and anemia as the most frequent severe AEs.^5^ Notably, toxicity appears milder than docetaxel-based combinations, making this option particularly attractive for patients who are unfit for chemotherapy.

In summary, treatment-related toxicity is a critical determinant in regimen selection, especially as treatment intensification becomes more common. Our NMA suggests that while adding a third agent may not significantly escalate the risk of severe AEs in an all-comer population, the low CoE warrants a cautious, patient-centered approach. Distinct toxicity profiles underscore the importance of matching the treatment to the patient's disease volume, biomarker status, and overall tolerability. Therefore, the therapeutic decision-making process in mHSPC is increasingly becoming a multidimensional task, requiring a nuanced understanding of both efficacy and risk.

Our study has several limitations. Combining rPFS and cPFS, which are not equivalent end points, may have introduced additional heterogeneity and resulted in estimates that do not fully reflect the true treatment effect. Differences in imaging modalities used for staging may also have influenced the results. Earlier studies primarily relied on conventional imaging, whereas more recent trials may have used more sensitive modalities, potentially leading to stage migration. Direct comparisons of key regimens (eg, docetaxel + ARPI + ADT vs ARPI + ADT) were unavailable, necessitating reliance on indirect contrasts in the presence of interstudy heterogeneity. The CoE was assessed using the CINeMA framework, which is recognized for its conservative approach. While this minimizes the risk of overestimating treatment effects, it may have resulted in more frequent downgrading of certainty compared with other methodologies. Evidence for PARP inhibitor–based, PSMA-targeted, and AKT inhibitor–based combinations was restricted to single trials with immature OS data. AE reporting was inconsistent, preventing comparative safety analyses across all effective regimens. De novo mHSPC subgroup data were unavailable for the newer combinations, precluding comparison with guideline-preferred triplet approaches. The exclusion of non-English studies may have introduced language bias.

## CONCLUSIONS

In this comprehensive NMA and SR, the combinations of docetaxel + ARPI + ADT and PARPi + ARPI + ADT showed clinically relevant but not statistically significant improvements in PFS for mHSPC. However, none of these intensified regimens demonstrated a statistically significant OS benefit over ARPI plus ADT in the all-comer population, although docetaxel + ARPI + ADT significantly improved OS in the high-volume subgroup. While our NMA did not identify a statistically significant increase in severe AEs for these triplets compared with ARPI + ADT, the low CoE and distinct toxicity profiles necessitate a cautious, patient-centered approach. These findings support a more selective, biomarker-driven strategy for treatment intensification, balancing potential oncological gains against toxicity. Given the reliance on immature OS data for newer targeted agents, further long-term follow-up remains essential to refine optimal treatment selection in the mHSPC landscape.
